# Metal–Peptide Complexes with Antimicrobial Potential for Cotton Fiber Protection

**DOI:** 10.3390/jfb14020106

**Published:** 2023-02-14

**Authors:** Stela Georgieva, Petar Todorov, Desislava Staneva, Petar Grozdanov, Ivanka Nikolova, Ivo Grabchev

**Affiliations:** 1 Department of Analytical Chemistry, University of Chemical Technology and Metallurgy, 1756 Sofia, Bulgaria; 2 Department of Organic Chemistry, University of Chemical Technology and Metallurgy, 1756 Sofia, Bulgaria; 3 Department of Textile, Leathers and Fuels, University of Chemical Technology and Metallurgy, 1756 Sofia, Bulgaria; 4 The Stephan Angeloff Institute of Microbiology, Bulgarian Academy of Sciences, 1113 Sofia, Bulgaria; 5 Faculty of Medicine, Sofia University “St. Kl. Ohridski”, 1407 Sofia, Bulgaria

**Keywords:** peptide, hemorphin, copper–peptide complexes, Cu(II) ions, cotton, antivirus protection

## Abstract

A study of the formation of copper (II) complexes with hemorphin peptide motifs in alkalic water solutions is presented. The effect of the peptide ligand on the complexing properties of the Cu (II) ion was quantified by giving the stoichiometry and stability of the complex compounds in the medium in which they are formed using voltammetric (cyclic) and spectral (UV-Vis and fluorimetric) analytical techniques. The resulting complexes were examined via IR spectroscopy to detect M-N and M-O oscillations and using the EPR approach in solution and in the solid phase to view the coordination and ligand binding regime. The possibility of the synergistic action of copper ions in the antivirus protection processes of cotton fibers coated in the same solvent with the newly obtained complex compounds was also investigated. One of the advantages is the formation of the complexes in an environment where the immobilization takes place, which contributes to increasing the efficiency of the process. The obtained results may serve as an aid for future more detailed biological studies of structure–activity relationships (SARs).

## 1. Introduction

Copper is a trace element of particular importance for proper functioning and maintaining the body’s function [1,2]. For example, copper ions are present in oxygen-using oxidases. At the same time, Cu also plays a signaling function that can influence cell behavior and metabolism. The complex binding of copper ions with peptide structures has promoted collagen production, increased vascular growth, and antioxidant capacity, and stimulated glucosaminoglycan production, supporting the skin’s self-repair processes [3]. As an activator of tissue remodeling, bound copper can also promote the growth, division, and differentiation of nerve cells, immune-related cells, and glomerular cells and stimulate the production of epidermal stem cell proliferation markers and integrin [4].

Tyrosine [5,6,7], histidine [6,7], glutamate/aspartate [1], and/or any permutation of these ligands are used as N, O, and S electron donor groups in peptide molecules for copper ion binding. Metal-based peptide antimicrobials exhibit their activity through multiple mechanisms, such as binding and cleavage of multiple nucleic acid targets and/or possible initiation of oxidative stress mechanisms [8,9]. They have potential for higher efficacy and broad-spectrum activity since more pathways would need to be circumvented to override their effect; treated with such molecules, microbes are less likely to develop resistance. The bioactivity of the compounds can be changed after chelation, both due to the differences in the conformation of the ligand resulting from the complexation and the nature of the metal ions [10].

It is known that protein molecules, particularly peptides, in the living organism are in solution; therefore, they cannot be viewed as a “static form” [11]. Their dynamic state is associated with fluctuations that occur due to the rotation of specific residues or parts of the molecule around simple bonds of the peptide chain built of amino acid residues. For conformational energy of the peptide molecule arising during this rotation to remain at a minimum, i.e., to lead to the “unfolding” of the molecule, stabilizing factors are needed, some of which may be metallic ions [1,12,13]. The involvement of metal ions in the peptide structure is very diverse [14]. For example, different types of biometal ions, such as Cu(II), Zn(II), Fe(II), Co(II), and Fe(III), could be bounded to some specific amino acid residues from the peptide skeleton and could play a crucial role in the structural organization and activity of enzymes and biologically active compounds [12,15].

**Figure 1:**
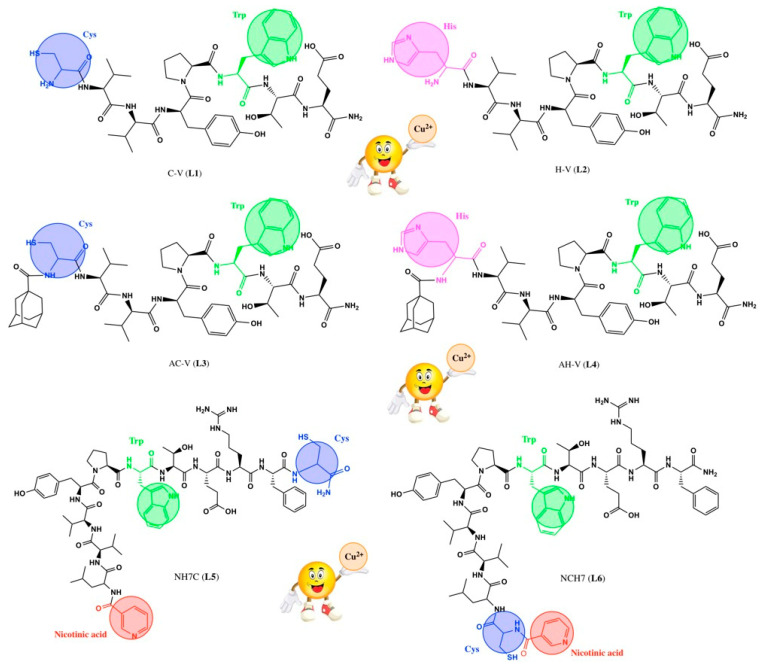
Structural formulas of the peptide ligands. The possible electron donor sites for complexation are given in color.

From what has been stated so far, we can conclude that the basis of understanding the binding of transition metals in proteins is the study of the complex-forming properties of amino acids and peptides with metal ions. In this regard, the main goal of the present study was to investigate the interactions between peptides with proven antimicrobial activity (structural formulas of the corresponding ligands are shown in Figure 1) and copper ions to evaluate the relationship between the coordination position in the peptide sequence of the hemorphin motif and metal ion binding properties. Hemorphin components VV-hemorphin-5 and VV-hemorphin-7 serve as the structural pillars of the examined ligands [16,17,18,19,20,21,22]. In our previous work, the hemorphin peptides were modified at the N and C terminals with different residues: Cys, Glu, His, 1-adamantanecarboxylic acid, and Niacin (Nicotinic acid) (see Figure 1) [23]. A similar modification leads to the optimization of the physicochemical properties of the hemorphin peptides and can increase their antimicrobial potency [23]. Copper ions are also known to have an affinity to coordinate with the nitrogen of the peptide bond to form cyan-violet complex compounds [24,25]. The present study examines how the sequences containing VV-hemorphin, together with the donor groups, may coordinate the copper(II) ions. It should also be mentioned that the binding of copper(II) ions depends also on the availability of binding sites (protein structure). The physicochemical properties of the obtained complexes with copper(II) ions were determined via spectroscopic methods (UV-Vis, fluorimetry, and EPR) and proved by voltamperometric techniques. Fluorimetric and UV-Vis measurements were used to calculate the stability constants and stoichiometry of the resulting complexes in an alkaline media, the environment in which the compounds were successfully deposited on cotton fibers. In addition, a comparative evaluation of the peptide ligands’ antiviral properties and of their copper complexes, both in solution and on cotton fibers, against the human respiratory syncytial virus (HRSV-S2) and Human adenovirus serotype 5 (HAdV-5), was undertaken.

## 2. Materials and Methods

### 2.1. Synthesis of the Peptides

The synthesis of the starting compounds used as ligands is described in detail in [23]. The solid-phase peptide synthesis by Fmoc chemistry was used to obtain the compounds. All chemicals and solvents used were of analytical grade and were purchased from Fluka or Sigma-Aldrich.

### 2.2. Physicochemical Characterization in Solution

#### 2.2.1. Spectral Measurements

##### Apparatus

The absorption spectra of the investigated compounds for the spectral characterization of the starting ligands and the obtained metallopeptide compounds were recorded in the UV-Vis region on a Varian-Cary spectrophotometer with 1 cm path length synthetic quartz glass cells. The fluorescence spectra of the peptide derivatives were recorded via a Cary Eclipse (Agilent, Santa Clara, CA, USA) spectrofluorometer in a range of 200–900 nm with a resolution of 0.5 nm and double-grating monochromators in excitation and emission. Potassium bromide (KBr) pellet and a Varian 660 FTIR spectrophotometer were used to obtain information on the IR spectrum of the investigated metallopeptide particles in a range 4000–500 cm^−1^ using Fourier-Transform Infrared Spectroscopy (FT-IR).

The EPR analysis in the current study was performed with a Bruker EMX Premium X EPR spectrometer operating in the X band at 9.4 GHz. For temperature variation, a thermoregulatory system, ER 4141 VTM, was used. The EPR spectra simulation was carried on Aniso-Spin program (Bruker Xenon software)

SEM-EDX equipment (SEM/FIB LYRA I XMU SEM (TESCAN)) was used for detection of copper (II) peptide complexes on textile fabrics, proving their presence. The analytical characteristics of the apparatus were as follows: tungsten heating filament; resolution—3.5 nm at 30 kV; accelerating voltage—200 V to 30 kV; EDX detector: Quantax 200 by BRUKER; spectroscopic resolution at Mn-Ka and 1 kcps 126 eV.

##### Solutions for UV-Vis and Fluorescence Analysis:

Water–methanol (1:1) standard solutions of the peptide compounds were used to perform the analyses for the preparation and characterization of the obtained complexes with concentrations as follows: 1.67 × 10^−3^ mol L^−1^ C-V; 1.30 × 10^−3^ mol L^−1^ AC-V; 1.17 × 10^−3^ mol L^−1^ NH7C; 1.37 × 10^−3^ mol L^−1^ AH-V; 1.85 × 10^−3^ mol L^−1^ H-V; 1.06 × 10^−3^ mol L^−1^ NCH7. Copper ions were introduced through a solution of Cu(NO_3_)_2_ × 3H_2_O with an initial concentration of 0.1020 mol L^−1^. All supporting solutions prepared with analytical-grade reagents (0.1 M ionic strength) are as follows: HCl + KCl (pH 1.25 ± 0.01), o-phosphoric acid/NaOH (pH 3.00 ± 0.01), CH_3_COOH/CH_3_COONa (adjusted to pH 4.52 ± 0.01, 5.45 ± 0.01 and 6.12 ± 0.01 with NaOH), NaH_2_PO_4_/Na_2_HPO_4_ (pH 7.21 ± 0.01), and Na_2_HPO_4_/NaOH (adjusted to 10.98 ± 0.01 and 12.00 ± 0.01 with NaOH).

To determine the pKa values of the peptide ligands, diluted aqueous solutions at different pHs (1.25 to 12) were prepared by diluting 0.100 mL of a standard solution of each peptide to a total volume of 4.00 mL with the respective supporting solution.

To determine the stoichiometry of the complexes at pH 10.98 ± 0.01, diluted solutions (with a total volume of 6.0 mL) were prepared so that, using Job’s approach, the total sum of the ligand and metal ion concentrations is constant for each molar series. For EPR analysis in solution (pH 10.98 and 7.21), series of solutions were prepared with an excess of peptide ligand (with molar ratio: n_Cu(II)_/n_peptide_ = 1/2) and concentration of copper ions in the solutions as follows: 2.01 × 10^−4^ mol L^−1^ Cu-C-V; 1.99 × 10^−4^ mol L^−1^ Cu-AC-V; 1.14 × 10^−4^ mol L^−1^ NH7C; 1.14 × 10^−4^ mol L^−1^ Cu-AH-V; 6.81 × 10^−5^ mol L^−1^ Cu-H-V; 1.14 × 10^−4^ mol L^−1^ Cu-NCH7. EPR studies of the complexes in solution were also carried out after dissolving amounts of the isolated complex compounds in 5.00 mL of phosphate buffer solution (pH 7.21). The concentrations of the organometal complexes are: 1.03 × 10^−4^ mol L^−1^ Cu-H-V; 8.01 × 10^−4^ mol L^−1^ Cu-NCH7; and 1.44 × 10^−4^ mol L^−1^ Cu-C-V.

#### 2.2.2. Electrochemical Measurements

For the electrochemical characterization of the compounds, a three-electrode voltammetric cell system connected to a 797 VA stand with experimental control and data acquisition connected to a Metrohm 797 VA trace analysis apparatus was used. A solid glassy carbon (GC) working electrode was connected in an electrochemical cell with Ag/AgCl, KCl as reference and platinum wire as auxiliary electrodes for cyclic voltammetric obtention of the analytical signals in medium of phosphate buffer (Na_2_HPO_4_/NaOH) with pH 10.98 ± 0.01. Each voltmaperogram was taken after gentle stirring and purging of 7.0 mL electrolyte solution containing aliquots of the analyte (100.0 to 800.0 μL) with pure nitrogen (99.999%) for 200 s at room temperature (25 °C).

### 2.3. Preparation of Complexes in Solid Phase

An accurately weighed amount of the starting ligands (0.0114 g L1, 0.01107 g L2, 0.0110 L3, 0.0150 g L4, 0.0120 g L5, and 0.0153 g L6) was added with continuous stirring to 5 mL of phosphate buffer medium (pH 10.98). After complete dissolution of the peptides, 25 µL of the aqueous standard solution of Cu(NO_3_)_2_ × 3H_2_O (0.1020 mol L^−1^) was added slowly, dropwise, to each solution. The solution was allowed to stir for 30 min, after which the precipitate was filtered and dried at room temperature (25 °C). Yield: Cu-L1: 67%, Cu-L2: 74%, Cu-L3: 59%, Cu-L4: 65%; Cu-L5: 53%, and Cu-L6: 55%.

ICP-OES was used to prove the presence of metal ions in solutions of the complex compounds. The estimated quantity matched the amounts of copper that were stoichiometrically added to the starting solutions to form the complexes. The ICP-OES spectra were recorded with a T Prodigy High-Dispersion ICP-OES spectrometer, Teledyne Leeman Labs, USA instruments, with operating conditions (a dual-view torch, cyclonic spray chamber) and concentric nebulizer with the following conditions (coolant gas 18 L min^−1^, auxiliary gas 0.5 L min^−1^, nebulizer gas 34 psi, RF power 1.2 kW, pump rate 1.2 mL min^−1^, sample uptake time 30 s, and integration time 40 s). High-purity Ar 99.999% supplied by SIAD BG was used to sustain plasma and as a carrier gas. Two-point background correction and three replicates were used to measure the analytical signal. A copper standard solution (‘Ultra scientific’, Lot: P00332) was used for calibration.

### 2.4. Coating and Fastness Test of Cotton Fibers with Alkaline Solutions of the Metallopeptide Complexes

Commercially purchased 100% cotton fabrics (140 g m^−1^, plain wave) were pre-washed in soapy water and left to dry at room temperature. The exhaustion method was used for immobilization of the metallopeptide complex. Pre-prepared alkaline solutions of the copper–peptide compounds with concentration ~1 × 10^−3^ mol L^−1^ were used to coat fabric samples by immersing them for 12 h. The temperature of the solution is kept at 50 °C for the first 6 h and at room temperature for the remaining 6 h. The volume of solutions was of liquor-to-goods ratio 1:5 [26].

The cotton fabrics were rinsed with distilled water and dried at room temperature. After deposition, the modified cotton fibers were tested for resistance by being washed in an alkaline soap solution according to established guidelines [27]; the fluorescence of the solutions was measured in Na_2_HPO_4_/NaOH (adjusted to 10.98 ± 0.01).

### 2.5. Virology

#### 2.5.1. Cytotoxicity Assay

Monolayer cells (grown in 96-well plates, Costar^®^, Corning Inc., Kennebunk, ME, USA) and inoculated with the research peptides at different concentrations (with 0.1 mL/well) were used for the cytotoxic experiments. Incubation of the cells was carried out for 48 h in at a temperature of 37 °C and 5% CO_2_. Standard procedures were carried out with a controlling purpose: (a) at each step of the protocol, a microscopic assessment was made; (b) the test substance was removed together with the supporting medium; (c) cells were washed with PBS; (d) culture medium containing 0.005% neutral red dye was added followed by 3 h incubation at 37 °C. This was followed by washing and desorption of the dye using glacial acetic acid and ethanol (0.15 mL/well 1% glacial acetic acid and 49% ethanol in distilled water). The result of the experiment was obtained by reading the optical density (OD) using a multiplate reader (Biotek Organon, West Chester, PA, USA) at 540 nm. Thus, 50% cytotoxic concentration (CC50) is determined at that concentration of the test substance that leads to 50% cell viability compared to control cells.

#### 2.5.2. Antiviral Activity Assay

The cytopathic inhibition (CPE) test was used to determine antiviral activity. To perform this test, 100 µL CCID50 per well (96-well plates containing 100 µL monolayer cells) was used, followed by one hour of virus adsorption with the addition of various concentrations of the test substance. Incubation of the cells was carried out for 48 h in an environment with a temperature of 37 °C and 5% CO_2_. The ability of live cells to accept neutral red and the ratio of the differences in the optical densities of the test samples to the virus control and the differences in the optical densities of the toxic control to the virus control are used to read this test. The selective index is determined by the ratio between the value of the 50% cytotoxic concentration and the 50% inhibitory concentration.

#### 2.5.3. Virucidal Assay

Virucidal activity of the tested substances (Cu-CV, Cu-HV, Cu-ACV, Cu-AHV, Cu-NH7C, and Cu-NCH7) was conducted against human adenovirus type 5 (HAdV5) and human respiratory syncytial virus (HRSV-S2) using the procedure described by Hossain’s group [28]. The applied concentrations of the complexes in the experiments are as follows: Cu-CV—20 µM/mL, Cu-HV—25 µM/mL, Cu-ACV—19 µM/mL, AHV—5 µM/mL, NH7C—7 µM/mL, and NCH7—14 µM/mL. Studies on virucidal activity were also carried out on cotton fabrics that were pre-impregnated with modified peptides, and for this purpose, identically sized pieces of textile (1 cm^2^) were used. The times for which these pieces are in contact with the virus (100 µL suspension) are 30 and 60 min, respectively. The same untreated fabric was used as a control.

## 3. Results and Discussion

### 3.1. Solution State Characterization

#### 3.1.1. Spectral Investigations: UV-Vis and Fluorimetry

**Figure 2:**
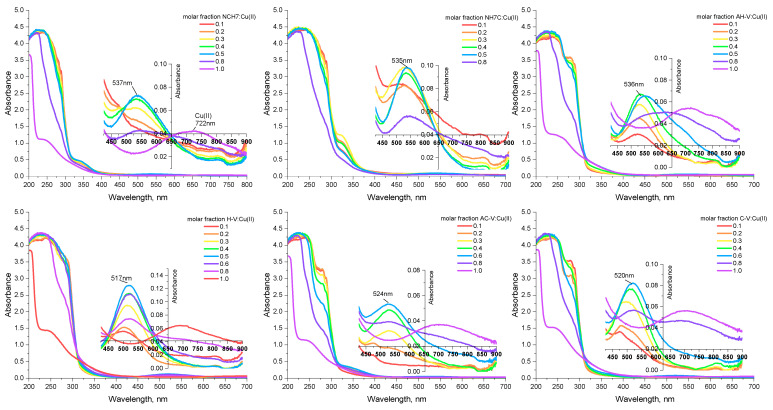
UV-Vis spectra of solutions (pH 10.98) with different peptide–metal ion molar ratio to prove composition and stoichiometry of the complex forms. The inset plots are magnified spectra in the visible region (where the complexes absorbed) of the same solutions. The concentrations of the stock solutions of Cu(II) and peptides for preparation of the molar fractions are as follows: Cu(II)-CV (C_C-V_ = C_C(II)_ = 1.67 × 10^−3^ mol L^−1^); Cu(II)-AC-V (C_AC-V_ = C_C(II)_ = 1.28 × 10^−3^ mol L^−1^); Cu(II)-H-V (C_H-V_ = C_C(II)_ = 1.85 × 10^−3^ mol L^−1^); Cu(II)-AH-V (C_AH-V_ = C_C(II)_ = 1.37 × 10^−3^ mol L^−1^); Cu(II)-NCH7 (C_NCH7_ = C_C(II)_ = 1.06 × 10^−3^ mol L^−1^); and Cu(II)-NH7C (C_NH7C_ = C_C(II)_ = 1.17 × 10^−3^ mol L^−1^).

**Figure 3:**
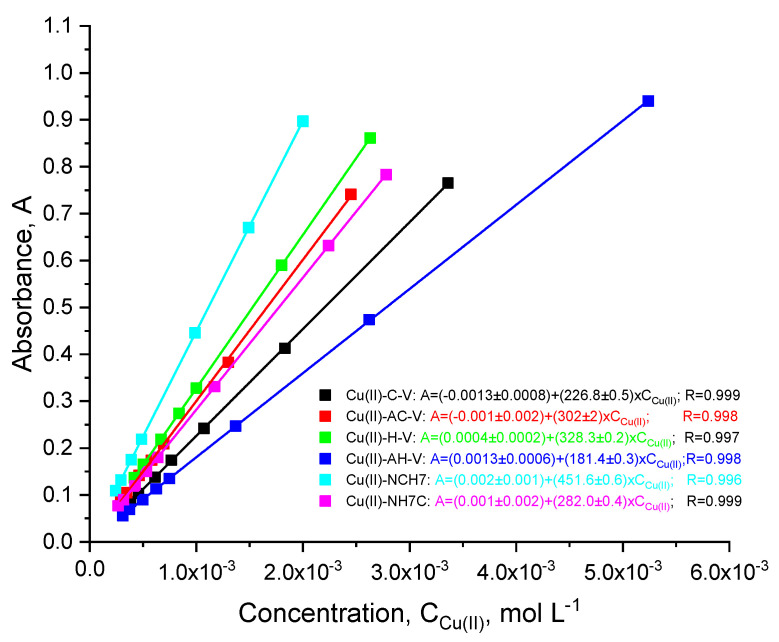
Plots of absorbance vs. concentration of Cu(II) ions in the metallopeptide complexes at pH 10.98. Absorbance was measured at 537 nm against an analytical blank.

The investigated peptide compounds showed biological activity, in which complexation can be considered as a potential solution for enhancing their antimicrobial properties. As a result of chelation, cellular defense mechanisms can be avoided without requiring chemical modification while preserving the native structure of the peptide. The following paragraph of this manuscript discusses the bioassays conducted on solutions of the investigated metallopeptides on certain pathogens. The peptide compounds used as complexing ligands are, as previously noted, various modifications of natural VV-hemorphin-5 and VV-hemorphin-7 [23]. Unlike AC-V, the H-V analog contains a heterocyclic basic amino acid His with a donor nitrogen atom from an imidazole moiety, favorable for complexation [23]. The rest of the peptide compounds differ among themselves by the variety of amino acid residues with free functional groups: -COOH, -SH, guanidine, and pyridyl and their different polarity, charge, hydrophobicity, etc., which they attach to the final peptide molecule. Thanks to the multiple coordination centers in the molecule of the studied peptides (Figure 1), the interaction process with copper(II) ions was initially followed by electronic spectroscopy in aqueous solutions at different pHs. It is known that peptide ligands are protonated differently depending on the pH of the medium. At pH > 8, the peptides are completely deprotonated [29]. The addition of the metal cations to the differently protonated peptide forms in M:L ratios from 1:10 to 2:1 induces the appearance of a UV-Vis absorption maximum in the visible part of the ligand spectrum in the presence of Cu(II) only in alkaline media (pH 10.98, Figure 2). The electronic spectra of the obtained complexes in phosphate buffer solution consist of high-intensity signals at 190–300 nm and a low-intensity, broadened band in the 520–530 nm interval (violet). The characteristic absorption band for amides is 190 nm, but usually, peptide compounds are recognized by their absorption around 280 nm, which is mainly due to the absorption of the side chains of the tyrosyl, tryptophan, partly phenylalanine amino acid residues [30]. Furthermore, the presence of the phenolic chromophore of tyrosine, present in the structure of all peptides, leads to the appearance of a shoulder at about 300 nm, due to the dissociation of the –OH group at higher pH values, causing a decrease in signal and bathochromic shift of the tyrosine maximum [31]. The asymmetric and broadened bands recorded in the peptide spectra in the visible region (at λ = 537 nm, Figure 2) are caused by d-d transitions in the metal ion influenced by the interaction with both nitrogen and oxygen donor atoms. A plot of absorbance versus Cu(II) ion concentration in solutions with an excess of the corresponding peptide ligand is presented in Figure 3. The obtained results show that the absorbance increased linearly with the concentration of Cu(II) ions at Cu(II)/peptide ratios ≥ 0.5. As described above, the only absorbing species at 537 nm is the copper–peptide complex (Figure 2), and the concentration increased linearly with the concentration of Cu(II). The obtained regression equations proved that Beer’s law is obeyed in the studied concentration interval of the complex (Figure 3). The molar absorptivity coefficient of the metal(II) complexes in the visible region corresponds to d-d transitions in the coordinated metal centers (Table 1) [32]. The preceding suggests the formation of mononuclear complex compounds with the composition (CuL) and (CuL2).

**Figure 4:**
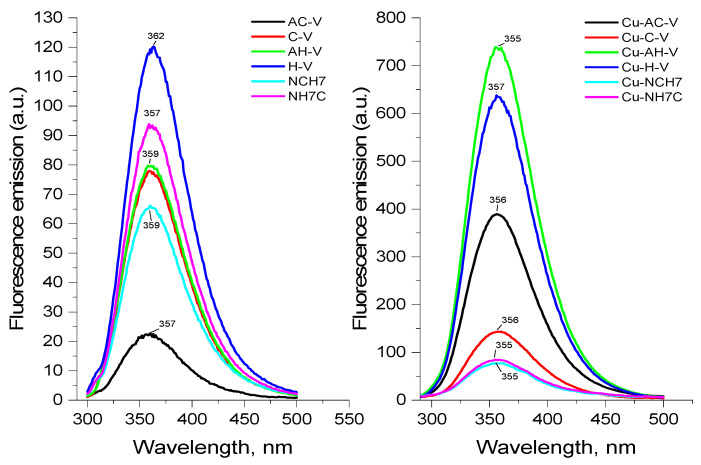
Fluorescence emission spectra of free and copper(II)-bound peptide compounds at equal concentrations of the ligands in the solutions (pH of the solution 10.98).

**Figure 5:**
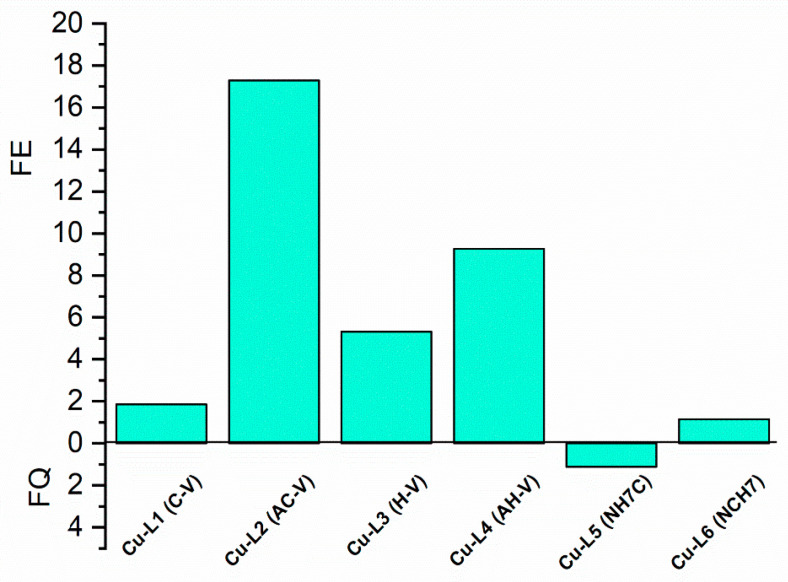
Effect of the Cu(II) ions on the fluorescence intensity of peptide ligands.

Fluorescence analysis is one of the most-sensitive methods for studying the interaction between the receptor fragment in a given molecule and its selective binding to the given substrate. As a result of this interaction, changes in the photophysical characteristics of the fluorophore fragment occur. A comparative examination of the solution fluorescence spectra of the free peptide forms and those bound to copper show that the character of the fluorophores of Trp and Tyr fluorescence band is of increasing intensity in the following sequence Cu-AH-V > Cu-H-V > Cu-AC-V > Cu-C-V > Cu-NCH7 and slightly quenched fluorescence for Cu-NH7C (L5), but unchanged in shape (Figure 4). Furthermore, the additional emission bands at the longer wavelengths were not observed, indicating the absence of any new emission species. The observations above suggested that the increased fluorescence of the peptides is enhanced by Cu^2+^ statically due to ground state complexation. The slight signal shift in the Cu(II)-containing particles, as well as the different intensity in their fluorescence spectra when compared to the uncoordinated peptide, indicates that the hemorphin derivatives undergo conformational changes during the complexation process. In Table 1, the Stokes shifts and percent increase in signal shift relative to the parent peptide derivatives are indicated. The most significant impact on the emission spectra, characterized by their bathochromic shift, is observed with the Cu-L4 complex. The fluorescence enhancement factors (FE, FE = I/I_0_) and quenching were determined from the ratio of the fluorescence intensity in the presence (I) and absence (I_0_) of metal cations. Figure 5 displays different values for all studied compounds; the highest ones pertain to Cu-AHV and Cu-ACV.

As can be seen, the fluorescence factor values depend on the nature of the receptor fragment in the peptide molecule directly relating to the different coordination sites of the copper ions, thus proving the complexation in solution. Copper ions are known to quench the fluorescence of solutions of Tyr and Trp [33]. It has been reported that the fluorescence of copper complexes with L-Tyr is restored after adding Cys [34] and histidine [35,36] to the reaction system. This leads us to consider that the presence of both amino acids in the peptide scaffold affects the emission signals of Tyr and Trp. Thus, it can be considered that the complexes are formed with the participation of the lone electron pair of the sulfur atom of the thiol group of cysteine for Cu-CV and Cu-ACV and that of the nitrogen atom of histidine of Cu-HV and Cu-AHV. This fact is also confirmed by the reported EPR analyses provided later in the manuscript. The effect of significantly increasing emission is not observed in the compounds Cu-NH7C and Cu-NCH7, where the nitrogen atoms in the pyridine (Figure 1) are possible coordination sites. A weak quenching of fluorescence is observed for the Cu-NH7C complex (Figure 4), owing to the chromophore’s weaker influence—the indole ring of Trp and the phenolic moiety of Tyr [37]. In L5 and L6 ligands, the amino acid phenylalanine is also present in the peptide chain, whose photosensitive chromophore part (phenolic moiety) is linked to cysteine at L5 and is the terminal amino acid at L6, respectively. In addition, the presence of a bulky electron-donating pyridine moiety would quench the fluorescence of the molecule, but at Cu-L6 there is an adjacent thiol group in copper coordination. The presence of phenylalanine also interferes with molecule folding, which, along with the “insertion” of copper at cysteine and pyridine, leads to the possibility of two or more ligands being coordinated. Job’s method demonstrated complex formation in a solution with a stoichiometry of 1:2. (Figure 5). However, with L5, cysteine is a terminal amino acid, but there is a slight fluorescence quenching, indicating that the pyridine is “free” to influence the chromophore’s electronic transitions from S to S0 to a greater extent. Job’s method demonstrated the formation of Cu-L5 complexes also with a stoichiometry of 1:2.

**Figure 6:**
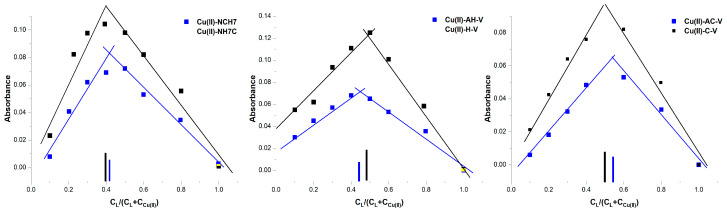
Plot of $C_{L}/\left(C_{L}+C_{Cu\left(II\right)}\right)=f\left(A\right)$ (Job’s plot) proving stoichiometry of copper peptide compounds. The sum of the concentration of the metal ion and the ligand in all solutions is the same.

**Figure 7:**
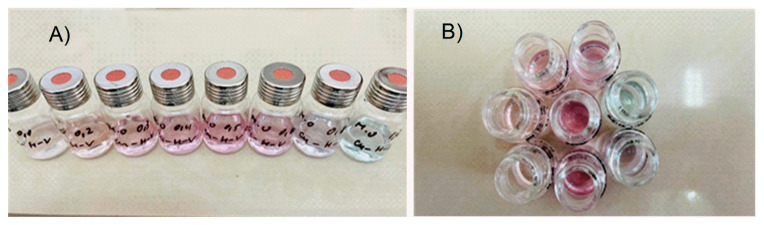
Solute color at different M:L2 molar ratios: (**A**) from left to right: 0.1, 0.2, 0.3, 0.4, 0.5, 0.6, 0.8, and 1.0; (**B**) when diluting solutions (**A**) twice.

**Figure 8:**
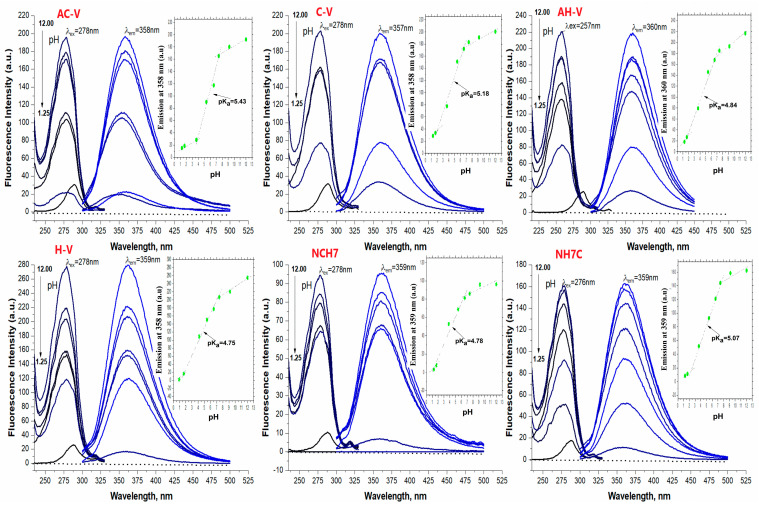
Excitation (in black)/emission (in blue) of peptide derivatives at different pH (from 1.25 to 12.0) and concentration of analytes as follow: 4.17 × 10^−5^ mol L^−1^ C-V; 3.23 × 10^−5^ mol L^−1^ AC-V; 2.93 × 10^−5^ mol L^−1^ NH7C; 3.43 × 10^−5^ mol L^−1^ AH-V; 4.63 × 10^−5^ mol L^−1^ H-V; 2.65 × 10^−3^ mol L^−1^ NCH7. The inset graphs are a plot of pH vs. emission intensity.

To obtain additional information on the composition of the newly acquired metallopeptide compounds and to confirm the number of coordinated ligands, Job’s method was applied using data from UV-Vis analyses (Figure 6). For this purpose, reaction mixtures were prepared in phosphate buffer solution (pH 10.98) containing ligand-deprotonated peptides and Cu(NO_3_)_2_ × 3H_2_O in ratios from 1:9 to 10:0. In each of the reaction mixtures, the volume and sum of the metal salt and ligand concentrations were kept constant. When the amounts of the ligand and metal ion are varied, violet-colored solutions are obtained (Figure 7), and with an excess of the metal salt, the color of the reaction mixture changes from violet to blue, indicating that a new type of complex particle is formed (Figure 6). Based on Job’s method, the M:L ratio was predominantly 1:2 for Cu-H-V, Cu-AH-V, Cu-NH7C, and Cu-NCH7 complexes and 1:1 for the remaining peptide forms (Figure 6). The experimentally observed evolution of d-d transitions in the electronic spectra of the violet-colored metallopeptides, as well as the results of the fluorescence analysis, indicate that the newly obtained compounds are in the exact assumed compositions: (CuL2) and (CuL). This stoichiometric form corresponds to copper peptide complexes known in the literature with a similar amino acid skeleton and simultaneously containing the coordination active histidine [38]. On the other hand, it is known that the stability of metallopeptide complexes increases proportionally with the number of amide bonds [39], because increasing the number of amide centers opens up the possibility of occupying all coordination sites in the coordination sphere of the metal ion. In addition, at pH 6 and above in the solution, predominated CuOH^+^ and Cu(OH)_2_ (aq.) form [40]. At pH 10.98, the product from the concentration of the metal ion (at ≈ 1 × 10^−5^ mol L^−1^) and OH^−^ ions could be described with the equation: $\left[C_{\mathrm{Cu}\left(\mathrm{II}\right)}\right]\cdot \left[C_{{\mathrm{OH}}^{-}}\right]=1.0\times 10^{-8}{\left({\mathrm{mol}\;L}^{-1}\right)}^{2}$, where [C] is the equilibrium concentrations of the copper and the OH^−^ ions, respectively. The obtained value is higher than the solubility constant (K_s_ = 10^−19.08^) which proves the formation of a precipitate of Cu(OH)_2_. The presence of a competitive complexation reaction and an environment of phosphate buffer solution does not allow for the precipitation of copper ions in the form of hydroxide, which, again, proves the binding of the peptides in a complex and the stability of the complex forms. The experimental values of the stability constants of the compounds and the degree of dissociation of the complexes are given in Table 2. For their calculation, the acidity constants of the peptide ligands were previously determined using data from the fluorescence analysis (Figure 8), and the values obtained are also given in Table 2. It can be seen that the obtained complexes are of approximately the same stability (Table 2) and the values are consistent with those of copper peptide compounds with similar structures reported in the literature [41,42].

#### 3.1.2. Cyclic Voltammetry

**Figure 9:**
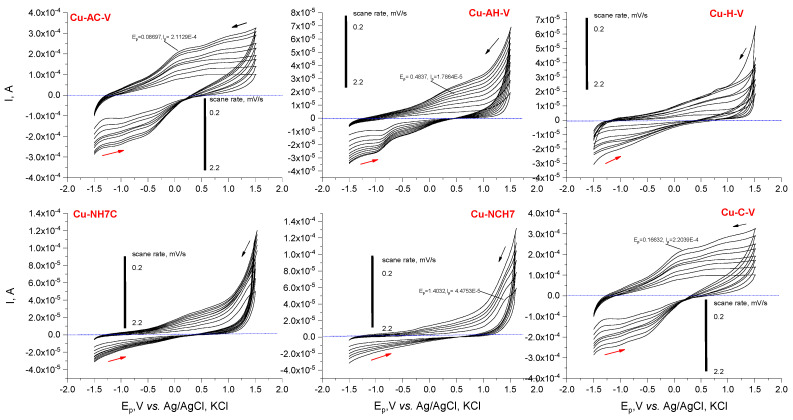
Plots of Ep, (V) vs. I, (A) in cyclic voltammetric determinations of the Cu(II)-peptide complexes at pH 10.98 and variation in scan rate from 0.2 to 2.2 mV s^−1^.

Voltammetry was also used as an analytical technique to prove the formation and stability of the obtained compounds in solution. In phosphate buffer solution (pH 10.98), the resulting complex compounds exhibit different electrochemical behavior than the unbound peptides. The anodic signals at ~0 V (for Cu-AC-V and Cu-C-V), ~0.5 V (for Cu-AH-V and Cu-H-V), and ~1.5 V (for Cu-NCH7 and Cu-NH7C) were observed due to the complex formation, the most sensitive being the signal for Cu-AC-V and Cu-C-V (Figure 9). A signal shift much more than 200–300 mV is observed due to His and Cys complexing, which were shown to oxidize irreversibly at −1.75 V in a slightly alkaline environment [23]. When copper ions are introduced into the solution, and due to the coordination of copper with the thiol and imidazole groups, a shift in the signals to the negative potentials is possible, which proves a connection in a stable complex [43]. Furthermore, the binding position of the copper appears to be necessary. When the copper coordinates to the cysteine without the presence of a pyridinyl moiety in the vicinity, a shoulder at about 0 V is observed, and when such a group is neighbored, a slightly intense maximum at 1.20 V is found at low scan rates. The variability in signal shape with increasing scan speed (only the intensity changes without shifting) suggests diffusion-controlled processes of charge transfer to the electrode surface and complex stability.

#### 3.1.3. EPR Analysis and IR Spectroscopy

**Figure 10:**
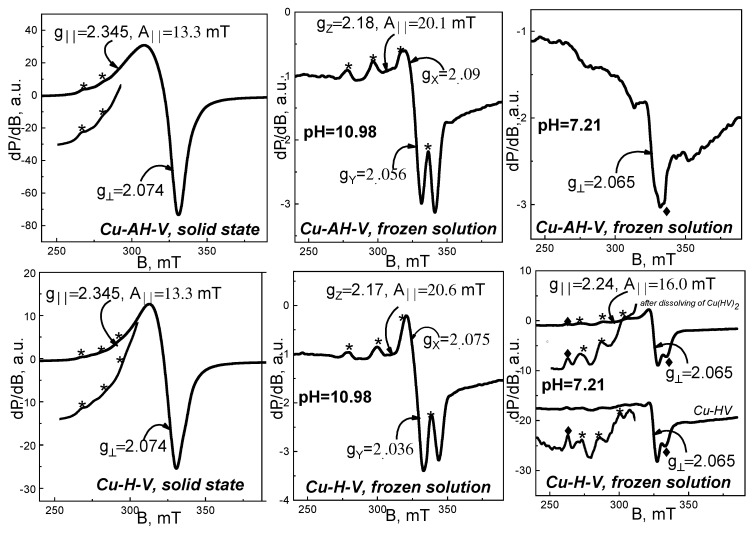
EPR spectra of organometallic compounds Cu(II)-AH-V and Cu(II)-H-V in solid state (**left**), frozen solution with pH = 10.98 (**middle**), and frozen solution with pH = 7.21 (**right**). The spectra are registered at 100 K for solid-state samples and of 120 K for frozen solution spectra. With asterisk * are labeled the lines of the hyperfine Cu^2+^ structures; With symbol ^♦^ are labeled the extra hfs lines at 262.5 and 335 mT of Cu^2+^ structures.

**Figure 11:**
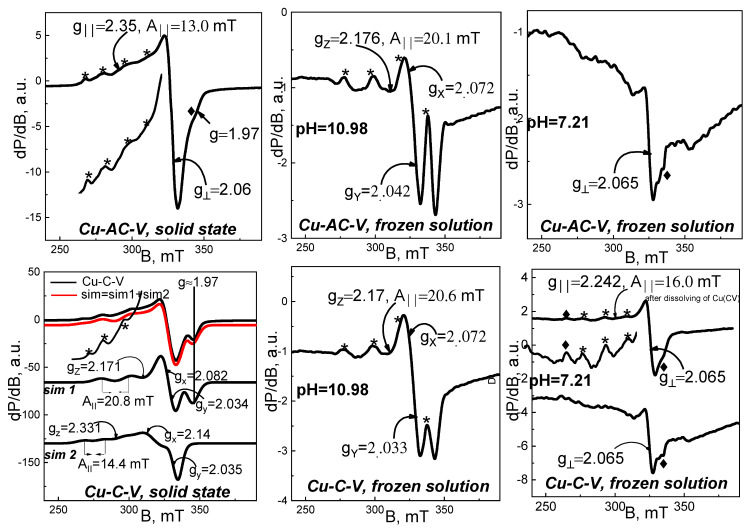
EPR spectra of organometallic compounds Cu^2+^-AC-V and Cu^2+^-C-V in solid state (**left**), frozen solution with pH = 10.98 (**middle**), and frozen solution with pH = 7.21 (**right**). The spectra are registered at 100 K for solid state samples and 120 K for frozen solution spectra. With asterisk * are labeled the lines of the hyperfine Cu^2+^ structures; With symbol ^♦^ are labeled the extra hfs lines at 262.5 and 335 mT of Cu^2+^ structures.

**Figure 12:**
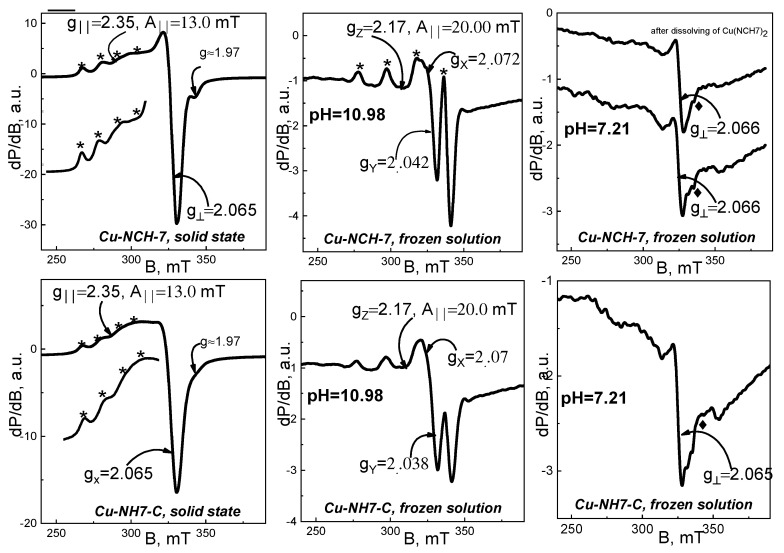
EPR spectra of organometallic compounds Cu(II)-NH-7 and Cu(II)-NCH-7 in solid state (**left**), frozen solution with pH = 10.98 (**middle**), and frozen solution with pH = 7.21 (**right**). The spectra are registered at 100 K for solid-state samples and 120 K for frozen solution spectra. With asterisk * are labeled the lines of the hyperfine Cu^2+^ structures; With symbol ^♦^ are labeled the extra hfs lines at 262.5 and 335 mT of Cu^2+^ structures.

An EPR analysis was performed on studied Cu^2+^ complexes, both in solid state and frozen liquid state at pH = 10.98 and pH = 7.21. The corresponding EPR spectra are shown in Figure 10, Figure 11 and Figure 12.

The spectra of solid-state samples were recorded in a temperature range 100–295 K, where the shape and position of all studied signals remained unchanged. As seen, the solid-state spectra of all complexes consist of asymmetric signals with parallel and perpendicular part, as g_‖_ > g_⊥_ > 2.0023. Superimposed on the parallel part are hyperfine structure lines, as their intensities and positions vary from sample to sample. The effective EPR parameters found for solid-state spectra are listed in Table 3. Based on the characteristics of solid-state signals, we could relate them to isolated Cu^2+^ ions in axially elongated tetragonal symmetry.

Solid-state EPR spectra of Cu(II)-AH-V and Cu(II)-H-V (Figure 10) are characterized by not-well-resolved HFS lines and by identical values of EPR parameters—g_‖_ ≈ 2.345, g_⊥_ ≈ 2.07, A_II_ = 13.3 mT, g_iso_ = 2.161. The nature of the four donor atoms in the equatorial plane of Cu^2+^ ions could be revealed using g_‖_- and A_II_- values. A relatively high value of g_‖_ accompanied by relatively low value of hyperfine structure constant, A_‖_, indicates coordination with oxygen atoms, while the low value of g_‖_ and high value of A_‖_ assume coordination with N atoms or N, S atoms. Therefore, for the complexes Cu(II)-AH-V and Cu(II)-H-V, coordination with O atoms is supposed.

Close examination of the increased parallel part of solid-phase Cu(II)-C-V spectrum indicates the existence of two quartets of HFS lines with different intensities. Using a simulation program, the signal was decomposed into two composite signals, described by the following EPR parameters—g_z_ ≈ 2.171, g_x_ ≈ 2.082, g_y_ ≈ 2.034, A_‖_ = 20.8 mT, g_iso_ = 2.079 (Figure 11, sim 1) for the more intensive one, and g_z_ ≈ 2.331, g_x_ ≈ 2.082, g_y_ ≈ 2.034, A_‖_ = 14.4 mT, g_iso_ = 2.146 for the less intensive (Figure 11, sim 2). The EPR parameters of the second composite signal (g_z_ ≈ 2.331) are similar to those of AH-V and H-V complexes, and suggest coordination of Cu(II)ions with oxygen donor atoms. The first composite signal (g_z_ ≈ 2.171) shows a distinctly low g-factor value and high A_hfs_ value. A signal with such parameters could be attributed to Cu(II) ions in coordination with four N atoms or in mixed coordination with both N and S atoms. Most likely, the amino group and the thiol group, or both groups, are involved in this coordination.

Unlike previously discussed complexes, in the Cu-AC-V solid-state spectrum, one well-resolved quartet of hyperfine structure lines could be observed (Figure 11). Taking into account this prominent quartet, we determined EPR parameters—g_‖_ ≈ 2.35, g_⊥_ ≈ 2.06, A_‖_ = 13 mT, g_iso_ = 2.156. They show similarity to those of Cu(II)-AH-V and Cu(II)-H-V, as well as to signal 2 in Cu-C-V spectrum, and so it can be concluded that Cu(II) ions in the Cu-AC-V complex are also coordinated with four O atoms. It should be mentioned that in the spectrum, an additional less-intense line is noticeable, with g = 1.97. Its position coincides with the fourth HFS line in the dominant spectrum of the Cu(II)-C-V complex (simulation 1). Therefore, it seems that part of the Cu(II) ions in Cu(II)-AC-V is coordinated to N atoms. The existence of a second quartet of HFS lines was confirmed by the extreme line roughness, visible in the increased parallel part of the spectrum.

The EPR spectra of solid-phase Cu-NH-7 and Cu-NCH-7 were established as main signals with EPR values—g_‖_ ≈ 2.35, g_⊥_ = 2.065, g_iso_ = 2.16 and A_‖_ = 13 mT (Figure 12). Similarly to Cu(II)-AH-V, the registered extra line with g = 1.97 (more prominent in Cu^2+^ NCH-7 complex) is a sign for the existence of a less-intensive signal, similar to signal 1 in the spectrum of Cu-C-V.

Taking into account the EPR results from studied solid-state organometallic complexes, it could be concluded that in the solid state, the coordination of Cu(II) ions depends on the type of ligand. The observed variation in the kind of EPR signals means that a number of donor atoms could be involved in the process.

EPR spectra of frozen solutions with pH = 10.98 and pH = 7.21 were registered at 120 K. Their spectra are demonstrated in Figure 10, Figure 11 and Figure 12. The EPR spectra of frozen alkaline solutions consist of one asymmetric signal with clearly resolved and intensive hyperfine structure lines in its parallel part. The EPR parameters for all studied complexes are distributed in a range of g_z_ ≈ 2.17–2.19, g_x_ ≈ 2.090–2.065, g_y_ ≈ 2.033–2.0056, and A_‖_ = 20.0–20.6 mT. As seen, these values are similar to those of signal 1 in the Cu-C-V spectrum. Therefore, in alkaline frozen solutions, one type of coordination is preferred, namely the coordination to N atoms. It could be concluded that a critical factor for the mechanism of coordination is the acidity of the solution. This statement was confirmed by the EPR analysis of frozen solutions with pH = 7.21. The EPR spectra of all solutions with pH = 7.21 show identical spectra that differ from the spectra of solutions with pH = 10.98. Although for part of these samples, it was not possible to determine g_‖_ and A_‖_, we can still summarize several observations: the value of g perpendicular for all signals at pH = 10.98 varies in a small range—2.063–2.069; the parameters g_‖_ and A_‖_ (g_‖_ ≈ 2.24, A_II_ = 16.0 mT) are common for Cu(II)-HV, Cu(II)-(HV)_2_, and Cu(II)-CV (pH = 7.21); in the spectra of mentioned samples, we noticed two extra hfs lines at 262.5 and 335 mT (designated by ♦), as the line at 335 mT is visible in the spectra of all studied in pH 7.21. Therefore, in neutral solutions, we established several different coordination for Cu(II) ions, as the main of them with g_‖_ ≈ 2.24, A_II_ = 16.0 mT refers to mixed coordination with N and O donor atoms. Unlike neutral solutions, in alkaline solutions, organometallic compounds with well-defined structures are stabilized.

**Figure 13:**
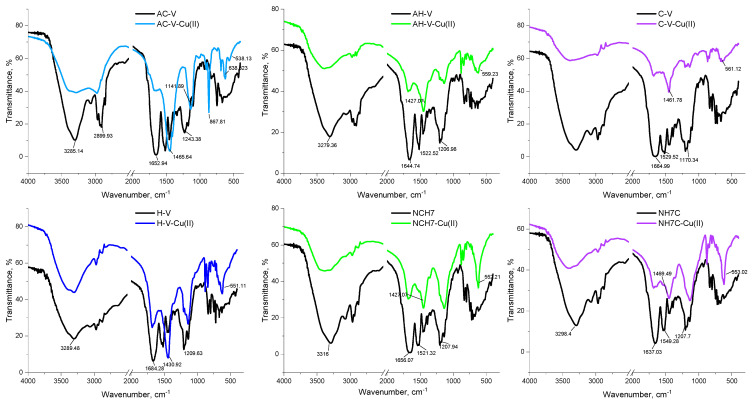
IR-spectra of the unbonded peptide (black line) and Cu(II)-peptide complexes (color lines).

**Figure 14:**
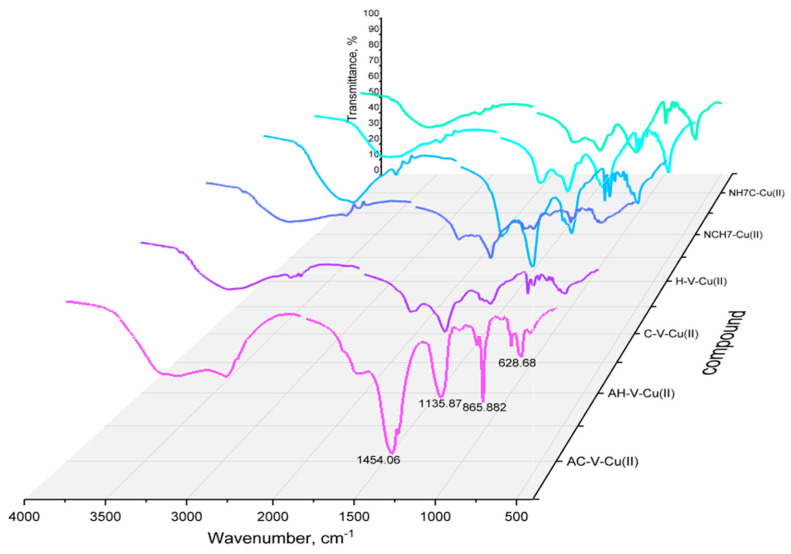
Comparative representation of IR spectra of Cu(II)-peptide complexes.

The IR spectra of the starting compounds and the resulting complexes with copper ions were compared with each other to confirm the complex form of the compounds after their isolation in pure form (Figure 13 and Figure 14). The characteristic bands for Amide I, II, and III corresponding to the groups νC=O and δNH were previously reported by Todorov’s group [23]. The possibility of incorporating copper ions into the molecule of peptide compounds is determined by the free coordination sites in the coordination sphere of the metal ion and by unobstructed oxygen and nitrogen atoms with which coordination is most likely to occur. During the formation of the complex forms, a change is observed both in the intensity (usually decreases) of the characteristic bands and in the qualitative appearance of the spectrum—displacement of bands and appearance/disappearance of others. After binding to the copper ions, a visible change in the energy of the characteristic bands is observed, especially in a range of 1800–400 cm^−1^. Comparing the IR spectrum of the “pure” peptides with that of the copper complexes, it can be seen that the latter lacks the band characteristic of some groups: NH_3_^+^ and COO^−^. In the area of 3300 cm^−1^, there is one stripe, characteristic of -NH_2_ group, connected in an intermolecular hydrogen bond, namely: νNH_2_ (~3285 and 3100 cm^−1^). Complexation does not affect the bands characteristic of alkyl substituents in the peptide molecule. After complex formation, the two -NH stretching vibrations can be observed, along with some typical deformation motions in this group. The energy shift observed for the carboxylate vibrations also confirms the involvement of this group in copper coordination. In the case of cysteine-containing peptides, a substantial decrease in the intensity of the S–H band (2890 cm^−1^ for AC-V and 2810 cm^−1^ for C-V) is observed, which can be attributed to the S–Cu interaction [44]. All metallopeptide IR spectra show decreasing characteristic bands of the carboxyl group at ~1650 cm^−1^, and increasing the peak at 1420–1470 cm^−1^ corresponds to a bending vibration of the -NH group, which proves the EPR thesis for coordination at -O and -N atoms. The normal coordinate analysis of the copper peptide complexes shows that one band in the 600–400 cm^−1^ range is due to the oscillation of the metal–ligand (M-N) bond, as follows: 538 cm^−1^ (Cu-AC-V), 559 cm^−1^ (Cu-AH-V), 561 cm^−1^ (Cu-C-V), 551 cm^−1^ (Cu-H-V), 553 cm^−1^ (Cu-NH7C), and 552 cm^−1^ (Cu-NCH7) (Figure 13).

**Figure 15:**
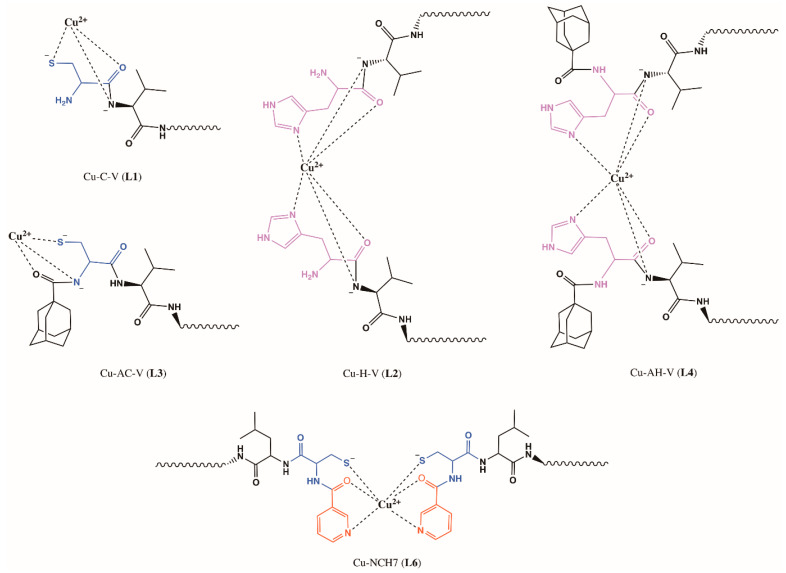
Schematic structure of Cu(II)-peptide complexes: Cu-C-V, Cu-AC-V, Cu-H-V, Cu-AH-V, and Cu-NCH7.

Based on the analysis of the complex compounds in solution and those isolated in pure form, we can present a presumed structure of the new Cu(II)-peptide forms (Figure 15).

#### 3.1.4. SEM Analysis

**Figure 16:**
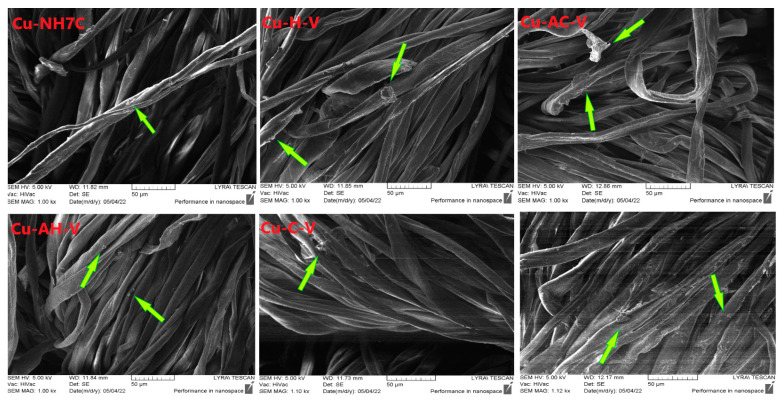
SEM images of cotton fabrics at 50 µm treated by copper peptides (57% in the solution). Green arrows show the presence of the complex compound on the treated cotton fibers.

**Figure 17:**
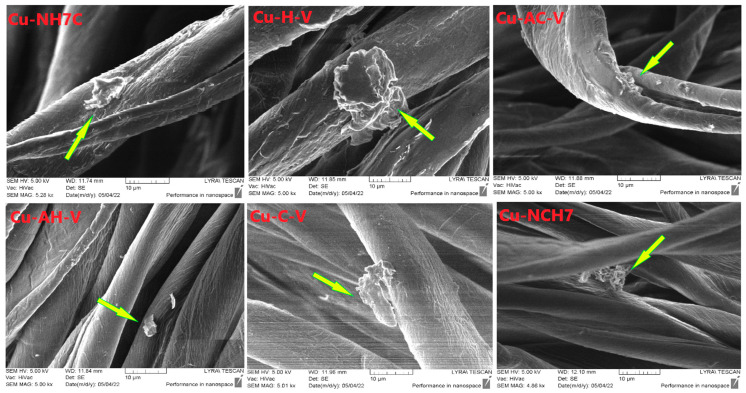
SEM images of cotton fabrics at 5 µm treated by copper peptides (57% in the solution). Yellow arrows show the presence of the complex compound on the treated cotton fibers.

**Figure 18:**
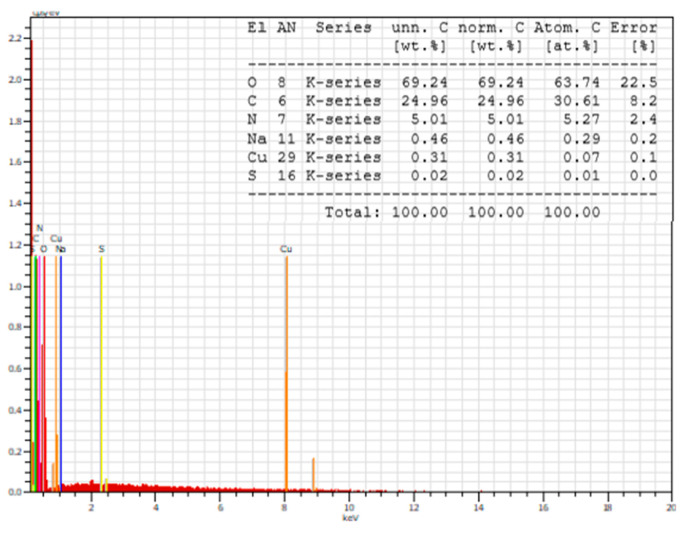
EDX spectrum of the Cu-C-V complex.

SEM analysis was used to observe the immobilization of peptide complexes on the fiber surface. Figure 16 shows images of cotton samples treated with each of the compounds. It is visible that the resulting coatings are uneven films. Larger particles are also observed on the surface of the fibers and are marked with yellow arrows. They are scattered unevenly and formed probably due to the processing method used. In Figure 17, showing photographs at a higher magnification, it is evident that these particles have an irregular shape and a layered structure. The elemental analysis carried out in part of the cotton surface shows the presence of the elemental characteristics of the compound (Figure 18).

### 3.2. Virological Activity

**Figure 19:**
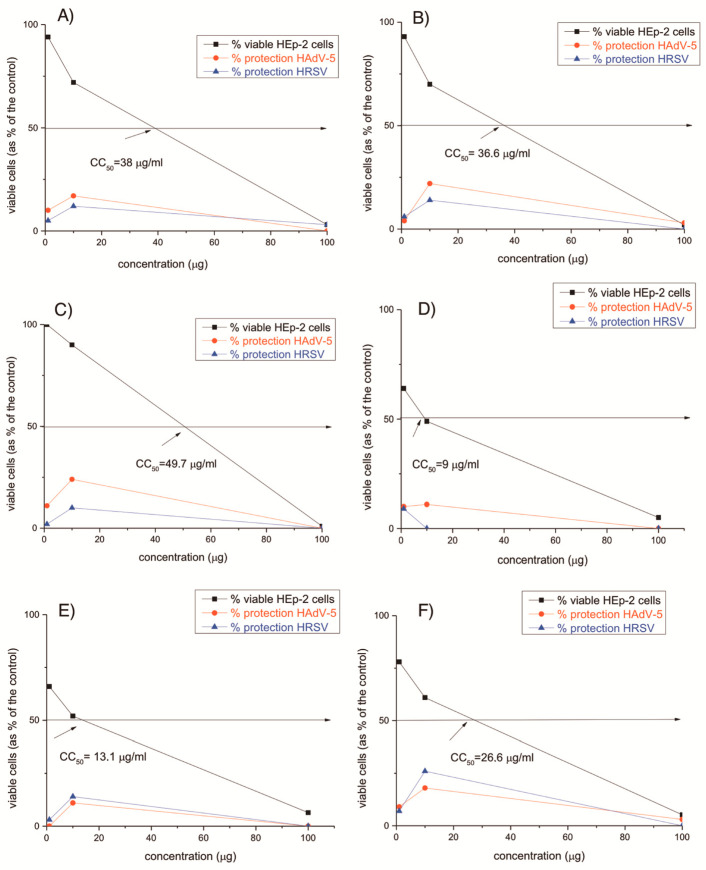
Antiviral activity (in red and blue) and cytotoxicity (in black) of: (**A**) Cu-C-V; (**B**) Cu-AC-V; (**C**) Cu-H-V; (**D**) Cu-AH-V; (**F**) Cu-NH7C; and (**G**) Cu-NCH7.

Antiviral activity of the newly synthesized copper(II)-peptide complexes is generally less pronounced compared to copper-free peptide molecules (Figure 19). The tested compounds Cu-CV, Cu-HV, Cu-ACV, and Cu-NCH7 showed weak virucidal activity against HRSV-S2, even after 30 min (Table 4). The virucidal activity of all tested peptides was found to be higher after 60 min against HRSV-S2. Cu-NH7C was the most potent complex (Δlog 1.2) compared to the rest of the tested samples. The investigated peptides described in the current article showed no virucidal activity against HAdV-5, regardless of treatment time. It is logical to assume that this is due to the more stable structure of this non-enveloped virus, which makes it resistant to chemical agents.

To investigate the virucidal effect against both HRSV-S2 and HAdV-5, cotton fabrics with peptide molecules immobilized on them were also investigated. Unfortunately, the virucidal effect was only observed against HRSV-S2 at 60 min (more than half a logarithm value) (Table 5).

All tested compounds did not show in vitro antiviral activity against HRSV-S2 and HAdV-5. The Cu-CV and Cu-HV complexes are more toxic than the other compounds. The reference substances and the results of the cytotoxicity tests of the compounds in HEp-2 cell culture are shown in Table 6. The difference in the biological activity of the complexes compared to the unbound peptides is probably due to the coordination of the copper to some of the active centers of the amino acid sequences of the peptide molecule. Probably for the same reasons, the virucidal effect of copper(II)–peptide complexes decreases and almost disappears. Ribavirin was used as the reference substance in this trial. This drug is part of the group of nucleoside analogues and has the ability to interfere with the synthesis of viral mRNA. The demonstrated cytotoxicity of the investigated peptide compounds is slightly higher compared to that of ribavirin; however, we should note that ribavirin is an oral drug, unlike the tested compounds, intended for processing and immobilization of fabrics.

## 4. Conclusions

In the present work, we propose a possibility for the complex formation between Cu(II) and hemorphin peptide derivatives in an aqueous solution at pH 10.98. Complete agreement with all spectroscopic data and voltammetric calculations confirmed that copper(II) complexes with peptides in aqueous solutions form stable complexes with 1:2 stoichiometry for Cu-H-V, Cu-AH-V, Cu-NH7C, and Cu-NCH7, and 1:1 for Cu-AC-V and Cu-C-V, weakly affecting the virucidal activity of human respiratory syncytial virus and adenovirus (HRSV-2) at 30 and 60 min, similarly to the starting peptide compounds. Complex formation with copper ions did not change their activity against human respiratory syncytial virus and adenovirus (HRSV-5), 30 and 60 min, with even a slight decrease in the virucidal activity at 30 min being observed compared to that of “free” peptide. Copper binding significantly affects the cytotoxicity of the molecule against HEp-2 cells, with a significant increase observed in Cu-H-V and Cu-C-V. The electrochemical behavior shows that the transport process/diffusion behavior of this Cu(II)-peptide system in solution is mainly controlled by the diffusion of the copper–peptide complexes without disturbing the integrity of the molecule in its interaction with a charged surface. The complex compounds isolated in pure form can be applied to modify cotton fabrics with an efficiency close to that of the unbound peptides. An advantage is the formation of the complexes in an environment in which the immobilization takes place, which contributes to increasing the efficiency of the process.

## Figures and Tables

**Table 1:** Photophysical characterizations of copper(II)–peptide complexes.

Compounds	λ_abs_ [nm]	ε, [L/(mol.cm)] at 272 nm	ε, [L/(mol.cm)] at 537 nm	λ^em^ [nm]	Stokes Shift [cm^−1^]	Percentage Increase (%) in Stokes Shift Relative to That of the Peptides Given in [23]
**Cu-L1 (C-V)**	272	5.40 × 10^6^	226	357	8753	54.65
**Cu-L2 (AC-V)**	270	6.90 × 10^6^	302	358	9104	54.65
**Cu-L3 (H-V)**	274	4.75 × 10^6^	328	359	8641	48.78
**Cu-L4 (AH-V)**	273	6.25 × 10^6^	181	360	8852	56.67
**Cu-L5 (NH7C)**	275	7.65 × 10^6^	282	359	8508	48.51
**Cu-L6 (NCH7)**	275	8.01 × 10^6^	451	359	8508	48.51

**Table 2:** Stability constants, degree of dissociation, and calculated acidity constants (logβ and pKa, respectively) of the studied copper(II) ion complexes and the studied ligands.

Species	CV (L1)	AC-V (L2)	H-V (L3)	AH-V (L4)	NH7C (L5)	NCH7 (L6)
pK ^a^						
pKa_(1–12)_	5.18	5.43	4.75	4.84	5.07	4.78
a ^b^						
Cu(II)	0.22	0.10	0.13	0.18	0.21	0.11
lgβ ^c^						
Cu(II)	4.92	5.72	5.31	5.18	5.09	5.65

^a^ acidity constant determined by plot of the function I = f(pH). ^b^ *a*- degree of dissociation: $a=\frac{C_{Cu}^{0}-C_{Cu\text{-}L}}{C_{Cu}^{0}}$; where *C_Cu-L_* is the molar concentration of the complexes at stoichiometric concentrations of M and L in the solution: $C_{Cu\text{-}L}=\frac{A}{\varepsilon }$; *A*- is the absorbance of the complexes at *C_Cu-L_* and ε is molar absorptivity at 537 nm. ^c^ stability constant of the complexes with stoichiometric: CuL_2_; $\beta =\frac{C_{Cu\text{-}L}}{{\left(C_{Cu}^{0}-C_{Cu\text{-}L}\right)}^{2}}$.

**Table 3:** The effective EPR parameters of organometallic compounds in solid state, frozen solution with pH = 10.98, and frozen solution with pH = 7.21.

Organometallic Compound	Frozen Solution pH = 7.21	Frozen Solution pH = 10.98	Solid State
**Cu(II)** **-AHV**	g_‖_—not determined g_⊥_~2.063 A_II_—not determined	g_z_ = 2.180 g_x_ = 2.090 g_y_ = 2.056 A_II_ = 20.1 mT	g_‖_ = 2.345 g_⊥_ = 2.074 A_II_ = 13.3 mT
**Cu(II)** **-HV**	*After dissolving the complex* g_‖_ = 2.24 g_⊥_ = 2.065 A_II_ = 16.0 mT	g_z_ = 2.170 g_x_ = 2.075 g_y_ = 2.036 A_II_ = 20.6 mT	g_‖_ = 2.345 g_⊥_ = 2.070 A_II_ = 13.3 mT
	*Complex forming in solution* g_‖_ = 2.24 g_⊥_ = 2.065 A_II_ = 16.0 mT	-	-
**Cu(II)** **-CV**	*After dissolving the complex* g_‖_ = 2.242 g_⊥_~2.065 A_II_ = 16.0 Mt *Complex forming in solution* g_‖_—not determined g_⊥_~2.065 A_II_—not determined	g_z_ = 2.170 g_x_ = 2.072 g_y_ = 2.033 A_II_ = 20.6 mT	g_z_ = 2.331 g_x_ = 2.14 g_y_ = 2.035 A_II_ = 14.4 mT g_z_ = 2.171 g_x_ = 2.082 g_y_ = 2.034 A_II_ = 20.8 mT
**Cu(II)** **-ACV**	g_‖_—not determined g_⊥_~2.067 A_II_- not determined	g_z_ = 2.176 g_x_ = 2.072 g_y_ = 2.042 A_II_ = 20.6 mT	g_‖_ = 2.32 g_⊥_ = 2.06 A_II_ = 13.0 mT
**Cu(II)** **-NH7C**	g_‖_—not determined g_⊥_~2.069 A_II_—not determined	g_z_ = 2.181 g_x_ = 2.065 g_y_ = 2.042 A_II_ = 20.0 mT	g_‖_ = 2.35 g_⊥_ = 2.065 A_II_ = 13.0 mT
**Cu(II)** **-NCH7**	g_‖_—not determined g_⊥_~2.067 A_II_—not determined	g_z_ = 2.190 g_x_ = 2.07 g_y_ = 2.038 A_II_ = 20.0 mT	

**Table 4:** Virucidal effect of Cu(II)-peptide complex systems against HRSV-S2 and HAdV-5 after 30 min/60 min.

Virus	Δlog 30 min						Δlog 60 min					
	Cu-CV	Cu-HV	Cu-ACV	Cu-AHV	Cu-NH7C	Cu-NCH7	Cu-CV	Cu-HV	Cu-ACV	Cu-AHV	Cu-NH7C	Cu-NCH7
**HRSV-2**	0.1	0.1	0.1	0	0	0.1	1.0	0.8	1.1	0.8	1.2	1.0
**HAdV-5**	0	0	0	0	0	0	0	0	0	0	0	0

**Table 5:** Virucidal effect of Cu(II)-peptide complex systems into cotton fabric against HRSV-S2 and HAdV-5 after 30 min/60 min.

Compound	Cytotoxicity
	CC_50_ (µM/mL) in HEp-2 Cells Peptide [23]/Cu-Peptide
**C-V/Cu-CV**	10.7/38
**H-V/Cu-HV**	12.2/49.7
**AC-V/Cu-ACV**	110/36.6
**AH-V/Cu-AHV**	106/9.0
**NH7C/Cu-NH7C**	170/13.1
**NCH7/Cu-NCH7**	139/26.6
**Ribavirin**	2058

**Table 6:** Cytotoxicity of newly synthesized Cu(II)-peptide complexes in HEp-2 cell culture.

Virus	Δlog 30 min						Δlog 60 min					
	Cu-CV	Cu-HV	Cu-ACV	Cu-AHV	Cu-NH7C	Cu-NCH7	Cu-CV	Cu-HV	Cu-ACV	Cu-AHV	Cu-NH7C	Cu-NCH7
**HRSV-2**	0.1	0	0	0	0	0	0.7	0.7	0.7	0.5	0.5	0.8
**HAdV-5**	0	0	0	0	0	0	0	0	0	0	0	0

## Data Availability

Samples of the compounds are available from the authors.
